# Laser Lead Extraction Complicated by Avulsed Tricuspid Subvalvular Apparatus with Severe Tricuspid Regurgitation

**DOI:** 10.19102/icrm.2020.110303

**Published:** 2020-03-15

**Authors:** Fotis N. Katsikeris, Calvin Craig, Colby Salerno, Mohammad Amin Kashef, Leng Jiang

**Affiliations:** ^1^Internal Medicine, Baystate Medical Center, Springfield, MA, USA; ^2^Division of Heart and Vascular Medicine, Baystate Medical Center, Springfield, MA, USA

**Keywords:** Laser lead extraction, lead extraction, transesophageal echocardiographytricuspid regurgitation, tricuspid valve

## Abstract

The use of laser lead extraction (LLE) to remove pacemaker or implantable cardioverter-defibrillator leads has become increasingly prevalent. This advanced technique has been shown to be highly effective and safe. We report a rare case of severe traumatic tricuspid regurgitation after LLE that led to death.

## Introduction

Transvenous lead extraction has become increasingly prevalent over the past few decades. The procedure when performed using advanced minimally invasive techniques has been shown to be highly effective and safe, with high success and low procedural complication rates, as reported in large multicenter registries^1–3^ and the real-world setting.^4^ However, complications are inevitable. Herein, we report a case of avulsed tricuspid subvalvular apparatus with severe tricuspid regurgitation (TR) after laser lead extraction (LLE) in a patient who was not a candidate for surgical repair and who died 10 days after the procedure.

## Case report

An 80-year-old malnourished female with a body mass index of 17.9 kg/m^2^ was referred for right ventricular (RV) lead extraction. Her pacemaker had been implanted 13 years previously, with a single RV lead (CapSureFix Novus model 5076; Medtronic, Minneapolis, MN, USA) for sick sinus syndrome. She had a complex medical history, including hypertension, hyperlipidemia, gout, insulin-dependent diabetes with neuropathy, end-stage renal disease on hemodialysis, severe anemia (hemoglobin: 8.4 g/dL; hematocrit: 27.6%), heart failure with preserved left ventricular ejection fraction, severe pulmonary hypertension (70 mmHg), chronic atrial fibrillation—for which she was taking Coumadin^®^ (Bristol-Myers Squibb, New York, NY, USA)—and peripheral vascular disease. She had also previously undergone a toe amputation for osteomyelitis.

The patient was admitted 15 days prior to the current case for abdominal pain. A computed tomography scan helped to raise the suspicion for a mycotic pseudoaneurysm of the lower descending thoracic aorta, which was confirmed by computed tomography angiography with noted rapid growth. Blood cultures within 24 hours of hospitalization grew methicillin-resistant *Staphylococcus aureus* (MRSA), and intravenous vancomycin was immediately initiated. To prevent life-threatening exsanguination of the pseudoaneurysm, an urgent endoluminal covered stent graft was put into place despite the risk of graft infection.

Regardless of antibiotic coverage (ie, vancomycin, piperacillin–tazobactam, and daptomycin), the MRSA bacteremia persisted even following the removal of the patient’s hemodialysis catheter, despite being afebrile with improved leukocytosis (white blood cell count decreased from 17.6 to 6.9 × 10^9^/L). Transthoracic and transesophageal (TEE) echocardiograms were then performed, which showed negative findings for valve or lead vegetation. However, the infection of her indwelling pacer remained of significant concern. The electrophysiologist was consulted, and it was agreed that the pacemaker and the single RV lead would be removed given her persistent MRSA bacteremia and the fact that she was not pacer-dependent per pacemaker interrogation.

**Figure 1:**
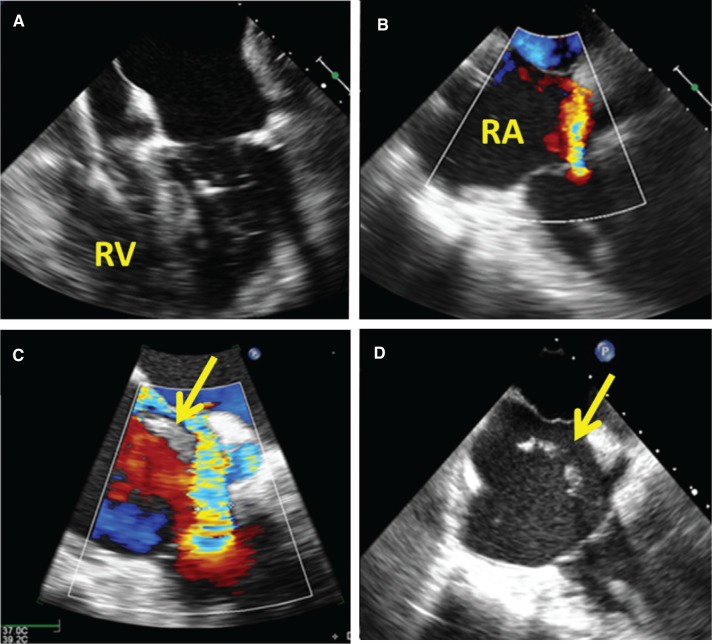
Intraoperative TEE images. **A and B:** Before the procedure, showing mild TR, with no evidence of vegetation. **C and D:** After the procedure, showing severe TR, with large jet width, spatial extension, and large proximal flow convergence region. There was avulsed papillary muscle and chordae tendineae that swung into the RA during systole (arrow). RA: right atrium; RV: right ventricle.

The extraction procedure proceeded following the reversal of anticoagulation. It was performed with a subclavian approach, under general anesthesia in the operating room, by an experienced operator. Intraoperative preprocedure TEE did not show evidence of vegetation; there was only mild TR **(Figures 1A and 1B)**. After the pulse generator was removed and the lead was freed from the scar tissue from the pocket, an LLD EZ locking stylet (Spectranetics Corp., Colorado Springs, CO, USA) was introduced and a 14-French laser sheath (Spectranetics, Colorado Springs, CO, USA) was employed, advanced to the tip of the lead to free the lead from any encapsulating scar tissue. While maintaining gentle traction, the single RV lead was successfully extracted without difficulty. However, the immediate postextraction TEE revealed severe TR, with evidence of avulsed papillary muscle and chordae tendineae **(Figures 1C and 1D)**. At that time, the patient appeared hemodynamically stable, with no evidence of hemopericardium or pericardial effusion on TEE. Considering her advanced age, frailty, multimorbidity, and persistent bacteremia, the team decided not to perform open repair of the tricuspid valve (TV) and instead continued with medical management.

**Figure 2:**
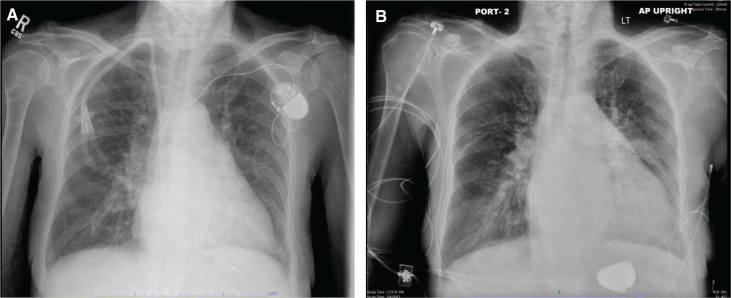
**A:** Preoperative X-ray. **B:** Postoperative X-ray.

In the subsequent days, the patient’s overall status deteriorated. She developed severe nausea, vomiting, noninfectious diarrhea, decreased oral intake, and cachexia with hypoalbuminemia (3.0 g/dL). There was a deterioration of her anemia (hemoglobin 7.0 g/dL; hematocrit: 23.4%) and metabolic panel (significant for pH: 7.35, calcium: 8.5 mg/dL, and phosphorus: 5.6 mg/dL). Her jugular venous distention was markedly elevated. She subsequently developed hypotension, was unable to tolerate hemodialysis, became unresponsive to fluid resuscitation, and had to be placed on norepinephrine infusion. The patient passed away 10 days after the extraction procedure. Her pre- and postprocedure chest X-rays **(Figure 2)** showed no pneumothorax or significant changes to her cardiac silhouette. The microbial cultures of the pacer lead and the hemodialysis catheter showed negative results.

## Discussion

The extraction of chronic indwelling pacemaker leads can be challenging due to many factors including time since implantation, systemic inflammation, and active infection. The use of LLE by way of emitting pulsed ultraviolet light and ablating the fibrotic adhesions typically facilitates lead extraction with high success rates. Associated life-threatening procedural complications, such as tearing of the superior vena cava or laceration of the RV or right atrium, have been significantly reduced using this method of lead extraction.^1–4^

TR associated with lead extraction has been considered less dramatic and has not been listed in the recently published National Cardiovascular Data Registry ICD registry.^4^ However, some reports have suggested an increase in TR with LLE (5.6%–9.1%),^5–7^ although the available data in the literature are conflicting.^8–10^ Some confounding factors have been reported, including issues with the study population (patient characteristics and risk factors), imaging tools, grading criteria for TR, and the protocols for laser use. In earlier studies,^5–7^ the causal role of the LLE in the occurrence of TR was more difficult to define since the use of LLE was only reserved for application following the failure of manual traction. The more recently published studies using only LLE^9,10^ reported no evidence of an increase in the severity of TR. Nonetheless, there were case reports of severe traumatic TR occurring after LLE and requiring surgery.^11–13^

It was postulated that the outer sheath and laser ablation can mechanically damage the valve apparatus, especially when there are severe fibrotic tissue adhesions between the lead and the TV apparatus. Therefore, ideally, such adhesions can be detected prior to the procedure, which may help in selecting an alternative inferior approach utilizing snares^14,15^ or even an open surgical approach to minimize valve damage. Currently, TEE is the imaging modality of choice for assessing valve anatomy and function. It is particularly useful in the assessment of the TV after lead extraction to identify new or worsening TR and the traumatic mechanism of TR, as shown in our case. With three-dimensional imaging, TEE can also demonstrate the detailed pathology of leaflet–lead adhesion; however, detecting adhesion between the lead and the subvalvular apparatus remains difficult because the subvalvular apparatus is imaged in the far-field and so is often covered by significant acoustic shadowing from the lead(s).

An important clue for lead–TV adhesion can be observed in the patient’s history, primarily the duration of the indwelling lead (13 years). Multiple relevant risk factors for LLE complications have also reported in the literature, including female sex; old age (≥ 75 years); low body mass index (< 25 kg/m^2^); the removal of two or more leads; and the presence of significant comorbidities such as advanced heart failure, end-stage renal dysfunction, anemia, and diabetes mellitus. Given these findings, our patient had a very high risk for adverse outcomes following the LLE procedure.

Traumatic TR has been considered a well-tolerated valvular abnormality, with only a small proportion of patients requiring valvular replacement or repair. Our patient had severe pulmonary hypertension with a pressure-overloaded RV. Her acutely developed severe traumatic TR, although hemodynamically stable initially, progressed rapidly to severe RV failure, which soon led to cardiointestinal syndrome, underfilled LV with low cardiac output, hypotension, and cardiogenic shock. She was deemed not a candidate for surgical repair given her significant comorbidities and died 10 days after the procedure.

This case underscores the importance of performing comprehensive preprocedure assessments. Although system infection, especially MRSA bacteremia, is the strongest indication for lead removal, risk stratification is critically important. All of the clinical risk factors for the procedure should be evaluated in each case before undertaking lead extraction, particularly in patients with multiple comorbidities. Our case may highlight the need for the introduction of a multifactorial risk assessment tool to risk stratify patients requiring LLE therapy. Strategies should be developed on a case-by-case basis. This case also highlights that a high-risk patient who is not a candidate for thoracotomy may be considered relatively contraindicated for lead extraction since life-threatening complications may arise.

## Conclusion

Avulsion of the tricuspid valve and/or subvalvular apparatus following LLE has not been widely reported; however, we should be mindful of this potential life-threatening complication in high-risk populations. Severe acute TR may not be tolerated in patients with underlying severe pulmonary hypertension. Therefore, comprehensive pre-LLE assessments including considering the risk factors and surgical candidacy should be systematically completed prior to LLE and strategies should be developed on a case-by-case basis. LLE in high-risk patients who are not candidates for thoracotomy may need to be considered as a relatively contraindicated procedure.
